# Mitochondrial DNA copy number variation, leukocyte telomere length, and breast cancer risk in the European Prospective Investigation into Cancer and Nutrition (EPIC) study

**DOI:** 10.1186/s13058-018-0955-5

**Published:** 2018-04-17

**Authors:** Daniele Campa, Myrto Barrdahl, Aurelia Santoro, Gianluca Severi, Laura Baglietto, Hanane Omichessan, Rosario Tumino, H. B(as). Bueno-de-Mesquita, Petra H. Peeters, Elisabete Weiderpass, Maria-Dolores Chirlaque, Miguel Rodríguez-Barranco, Antonio Agudo, Marc Gunter, Laure Dossus, Vittorio Krogh, Giuseppe Matullo, Antonia Trichopoulou, Ruth C. Travis, Federico Canzian, Rudolf Kaaks

**Affiliations:** 1 Department of Biology, University of Pisa, Pisa, Italy; 2 Division of Cancer Epidemiology, German Cancer Research Center/Deutsches Krebsforschungszentrum (DKFZ), Im Neuenheimer Feld 280, 69120 Heidelberg, Germany; 3 Department of Experimental, Diagnostic and Specialty Medicine (DIMES), University of Bologna, Bologna, Italy; 4 Centre de Recherche en épidémiologie et Santé des populations (CESP), Faculté de médecine - Université Paris-Sud, Faculté de médecine - Université de Versailles Saint-Quentin-en-Yvelines (UVSQ), Institut national de la santé et de la recherche médicale (INSERM), Université Paris-Saclay, 94805 Villejuif, France; 5 Institut Gustave Roussy, F-94805 Villejuif, France; 6 Department of Clinical and Experimental Medicine, University of Pisa, Pisa, Italy; 7 Cancer Registry and Histopathology Department, “Civic - M.P. Arezzo” Hospital, Azienda Sanitaria Provinciale Di Ragusa, Ragusa, Italy; 8 Department for Determinants of Chronic Diseases (DCD), National Institute for Public Health and the Environment (RIVM), PO Box 1, 3720 BA Bilthoven, The Netherlands; 9 Department of Epidemiology and Biostatistics, The School of Public Health, Imperial College London, St. Mary’s Campus, Norfolk Place, London, W2 1PG UK; 10 Department of Social & Preventive Medicine, Faculty of Medicine, University of Malaya, Pantai Valley, 50603 Kuala Lumpur, Malaysia; 11 Department of Epidemiology, Julius Center for Health Sciences and Primary Care, University Medical Center Utrecht, Utrecht, The Netherlands; 12 Department of Epidemiology and Biostatistics, Medical Research Council-Public Health England (MRC-PHE) Centre for Environment and Health, School of Public Health, Imperial College London, London, UK; 13 Department of Community Medicine, Faculty of Health Sciences, University of Tromsø, The Arctic University of Norway, Tromsø, Norway; 14 Department of Research, Cancer Registry of Norway, Institute of Population-Based Cancer Research, Oslo, Norway; 15 Department of Medical Epidemiology and Biostatistics, Karolinska Institutet, Stockholm, Sweden; 16 Genetic Epidemiology Group, Folkhälsan Research Center, Helsinki, Finland; 17 Department of Epidemiology, Regional Health Council, Biomedical Research Institute of Murcia (IMIB-Arrixaca), Murcia, Spain; 18 Consorcio de Investigación Biomédica en Red de Epidemiología y Salud Pública (CIBERESP), Madrid, Spain; 19 Department of Health and Social Sciences, Universidad de Murcia, Murcia, Spain; 20 Escuela Andaluza de Salud Pública, Instituto de Investigación Biosanitaria (ibs.GRANADA), Hospitales Universitarios de Granada/Universidad de Granada, Granada, Spain; 21 Unit of Nutrition and Cancer, Bellvitge Biomedical Research Institute (IDIBELL), Catalan Institute of Oncology, L’Hospitalet de Llobregat, 08908 Barcelona, Spain; 22 International Agency for Research on Cancer, Lyon, France; 23 Epidemiology and Prevention Unit, Fondazione Istituto di Ricovero e Cura a Carattere Scientifico (IRCCS)-Istituto Nazionale dei Tumori, Via Venezian, 120133 Milan, Italy; 24 Department Medical Sciences, University of Torino and Human Genetics Foundation (HuGeF), Torino, Italy; 25 Hellenic Health Foundation, 11527 Athens, Greece; 26 Cancer Epidemiology Unit, Nuffield Department of Population Health University of Oxford, Oxford, OX3 0NR UK; 27 Genomic Epidemiology Group, German Cancer Research Center/Deutsches Krebsforschungszentrum (DKFZ), Heidelberg, Germany

**Keywords:** Mitochondrial copy number, Telomere length, Breast cancer, Cancer epidemiology

## Abstract

**Background:**

Leukocyte telomere length (LTL) and mitochondrial genome (mtDNA) copy number and deletions have been proposed as risk markers for various cancer types, including breast cancer (BC).

**Methods:**

To gain a more comprehensive picture on how these markers can modulate BC risk, alone or in conjunction, we performed simultaneous measurements of LTL and mtDNA copy number in up to 570 BC cases and 538 controls from the European Prospective Investigation into Cancer and Nutrition (EPIC) cohort. As a first step, we measured LTL and mtDNA copy number in 96 individuals for which a blood sample had been collected twice with an interval of 15 years.

**Results:**

According to the intraclass correlation (ICC), we found very good stability over the time period for both measurements, with ICCs of 0.63 for LTL and 0.60 for mtDNA copy number. In the analysis of the entire study sample, we observed that longer LTL was strongly associated with increased risk of BC (OR 2.71, 95% CI 1.58–4.65, *p* = 3.07 × 10^− 4^ for highest vs. lowest quartile; OR 3.20, 95% CI 1.57–6.55, *p* = 1.41 × 10^− 3^ as a continuous variable). We did not find any association between mtDNA copy number and BC risk; however, when considering only the functional copies, we observed an increased risk of developing estrogen receptor-positive BC (OR 2.47, 95% CI 1.05–5.80, *p* = 0.04 for highest vs. lowest quartile).

**Conclusions:**

We observed a very good correlation between the markers over a period of 15 years. We confirm a role of LTL in BC carcinogenesis and suggest an effect of mtDNA copy number on BC risk.

**Electronic supplementary material:**

The online version of this article (10.1186/s13058-018-0955-5) contains supplementary material, which is available to authorized users.

## Background

Mitochondria are responsible for essential functions in the eukaryotic cell, such as energy metabolism, calcium homeostasis, and apoptosis [1, 2]. Mitochondria are also responsible for the generation of ROS, which is a by-product of energy metabolism. They possess their own genome (mitochondrial DNA [mtDNA]), and in each eukaryotic cell, there can be hundreds or even thousands of copies of their genomes [3]. mtDNA copy number reflects the number of mitochondria within each cell, and this number is maintained within a constant range in order to sustain the energetic needs of the cell [4]. Although mtDNA copy number differs by cell type, it has been observed that there is a good correlation between the number of copies of mtDNA across different cell types within the same individual [5]. Thus, measuring mtDNA copy number in blood has been proposed as a reliable and noninvasive method for estimating the average number of mitochondria in [5].

Over the last decade or so, several reports have suggested that mtDNA content measured in blood may be a risk factor for various diseases, including metabolic diseases, diabetes, obesity, neurodegenerative diseases, and cancer [5–13]. The majority of these studies suggest that high mtDNA copy numbers are associated with increased disease risk. One possible explanation for the association of mtDNA copy number with chronic disease risk is oxidative stress. The mitochondrial genome is particularly prone to DNA damage and mutations caused by oxidative stress, and mtDNA mutations are associated with increased mtDNA copy number as a possible compensatory mechanism to cope with mitochondrial dysfunction.

The study of mtDNA copy number variation in breast cancer (BC) is of particular interest, given the role of oxidative stress in the disease’s etiology [14–17]. Only four studies have investigated the role of mtDNA copy number in BC, and these suggested an association between high copy number and increased risk of developing the disease [5, 9, 13, 18]. All of these studies were focused entirely or predominantly on estrogen receptor (ER)-positive BC.

mtDNA deletions are a heterogeneous assortment of mtDNA mutations in terms of length and position and represent the major contributor to mitochondrial dysfunction [19]. Along the mtDNA circle, there are hotspot positions for deletions, and 90% of these include the loss of the nicotinamide adenine dinucleotide dehydrogenase 4 (*ND4*) sequence [20]. The most frequent deletion is the “common” deletion of 4977 bp, including the *ND4* sequence, which causes primary mitochondrial diseases but has also been associated with age-related disease susceptibility, such as cancer, muscle atrophy, and neurodegeneration [21, 22]. Moreover, in a recent study, Nie and colleagues reported that the common deletion in the mitochondrial genome could increase the risk of developing BC [23].

Besides oxidative stress, mitochondrial dysfunction (measured as decreased mitochondrial mass and energy production) recently has been associated with telomere attrition and shortening, and mtDNA copy number has been associated with leukocyte telomere length (LTL) [24]. A possible mechanistic link between telomere length and mitochondrial function has been proposed through the peroxisome proliferator-activated receptor-γ pathway [25]. To date, a number of studies have examined LTL in surrogate tissues in relation to cancer risks. However, the results have been inconsistent, showing positive, inverse, or null associations between telomere length and cancer risk, with the majority reporting that shorter telomere length increases the risk (reviewed in [26–29]). In relation to BC risk, studies have shown conflicting results [26, 29]; however, none of these studies were focused specifically on ER− BC risk.

To date, LTL and mtDNA copy number and deletion levels have been examined mostly as independent contributors to cancer risk. Recent studies have indicated that LTL and mtDNA copy number are positively correlated in healthy individuals, in pregnant women, and in patients with psychological disorders [24, 30, 31]. Moreover, very recently, it has been demonstrated that LTL and mtDNA copy number are correlated in intestinal gastric cancer [32]. Hence, there is emerging evidence that the markers are linked biologically and that their joint measurement may increase their predictive value for cancer risk. Therefore, to gain a more comprehensive picture of how the markers mentioned above can modulate BC risk, alone or in conjunction, we measured mtDNA copy number, LTL, and mtDNA deletions in the context of the European Prospective Investigation into Cancer and Nutrition (EPIC) cohort. In addition, given the fact that ER− BC has been greatly understudied for these markers, we collected a fairly large number of them (*n* = 251).

## Methods

### Study population: the EPIC cohort

The EPIC cohort has been described in full detail elsewhere [33]. Briefly, EPIC consists of about 520,000 healthy volunteers, aged 35–69 years, who were recruited between 1992 and 2005 in 10 European countries. All EPIC study subjects provided anthropometric measurements (height, weight, and waist and hip circumferences) and extensive standardized questionnaire information about medical history, diet, physical activity, smoking, and other lifestyle factors. The women also answered questions about menstrual and reproductive history, hysterectomy, ovariectomy, and use of exogenous hormones for contraception or treatment of menopausal symptoms. About 260,000 women and 140,000 men provided a blood sample, which was split into aliquots of plasma, serum, buffy coat, and erythrocytes and stored frozen for later laboratory analyses.

Cases of cancer occurring after recruitment into the cohort and blood donation are identified through local and national cancer registries or by a combination of contacts with national health insurance and/or active follow-up through the study subjects or their next of kin. Cancer incidence data are classified according to the International Classification of Diseases, Tenth Revision (ICD-10), system. Incident cases of BC were identified as first occurrence of primary invasive tumors, ICD-10 code C50, occurring among women who had no previous diagnosis of cancer. Since 2001, an expanding series of nested case-control studies have been conducted on hormonal, metabolic, and other blood-based risk factors for BC [34–37], as well as on genetic determinants [14, 38–45]. For these latter studies, the cases and controls were not individually matched; however, care was taken to select the controls randomly from the cohort to avoid selection bias. Because there were insufficient amounts of biological material (extracted buffy coat DNA) for some of the selected controls, we retained slightly uneven numbers of invasive BC cases. We had measurements on telomere length for 570 controls and 533 cases, measurements on mtDNA copy number on 548 controls and 522 cases, and measurements on mtDNA deletions on 539 controls and 519 cases. This study was approved by the ethical review board of the International Agency for Research on Cancer.

### Sample preparation and DNA extraction

All blood specimens were kept frozen in liquid nitrogen at the International Agency for Research on Cancer. DNA was extracted from blood samples on an Autopure instrument (Qiagen, Hilden, Germany) with Puregene chemistry (Qiagen). All of the samples were extracted using the same method and in batches of 96 to avoid any possible bias due to sample handling. The order of DNA from cases and controls was randomized on PCR plates, and each batch cases and controls was chosen to be from the same study center.

### LTL and mtDNA copy number/deletion measurement

LTL was measured by real-time qPCR as the number of copies of telomeric repeats compared with a single copy gene (*RPLP0*) used as a quantitative control. For each sample plate, a standard curve was prepared by using a serially diluted reference DNA (range 0.468–30 ng/μl). The relative length of telomeres (T) compared with the control gene (S) was calculated as the ratio T/S. All the samples were analyzed in triplicate. mtDNA copy number and deletions were also analyzed by qPCR. The mtDNA/nuclear DNA content was assessed using specific primers designed for the mitochondrial *ND1* gene and the nuclear β-actin gene. The ratio between these two genes (*ND1*/β-actin) identifies the relative mtDNA copy number [46]. To quantify mtDNA deletions, primers for the mitochondrial gene *ND4*, located in the major arch of the mtDNA where deletion frequently occurs, were used in combination with those of the mitochondrial gene *ND1*, which is rarely deleted. The mtDNA deletion level is reported as the ratio between *ND4* and *ND1* [47] and thus represented by the quantification of the nondeleted fraction of mtDNA (*ND4*) vs. the total amount of mtDNA (*ND1*). As for the LTL measurement, for the analysis of mtDNA, a standard curve was also used in each plate. Four samples were included in all plates for all the assays for quality control.

### Longitudinal assessment of LTL and mtDNA copy number

To assess the reliability of the measure of mtDNA content and LTL over time, we compared the measurements of these two markers obtained from samples collected 15 years apart from a set of 96 samples of healthy individuals randomly selected from among the EPIC Heidelberg cohort. We calculated intraclass correlations (ICCs) by dividing the between-person variance by the total variance (sum of between- and within-person variances) [48]. ICCs ≥ 0.75 are considered an indicator of excellent reliability; ICCs between 0.51 and 0.74, good reliability; ICCs between 0.40 and 0.50, fair reliability; and ICCs ≤ 0.40, poor reliability. This threshold has been selected according to a similar study done on 100 EPIC subjects in which researchers evaluated the reliability of serum metabolite measurements [49].

### Statistical analysis

Quantitative measurements of LTL and mtDNA copy number were log-transformed to obtain variables with an approximately normal distribution. The relationships of age, smoking, and body mass index (BMI) on the biomarkers (LTL and mtDNA copy number/deletion) were examined by linear regression, adjusting for plate number, center, and case-control status. Correlation between the biomarkers (LTL vs. mtDNA copy number, LTL vs. mtDNA, mtDNA copy number vs. deletion) was examined by calculating Pearson’s correlation coefficients and corresponding *p* values.

To examine the relationships of LTL and mtDNA copy number in relation to BC risk, we used unconditional logistic regression to obtain ORs and 95% CIs adjusted for age, center, smoking status, BMI, and plate. We also performed stratified analyses by ER status. LTL and mtDNA copy number were analyzed as continuous variables, and as ordinal variables using quartile cutpoints for the variable distributions among the controls. To investigate the aggregated effects of mtDNA copy number together with LTL for each individual, the quartiles of the log-transformed values of the markers were added together, creating a score with values ranging from 2 to 8. The score was investigated as a continuous variable accounting for age, center, and plate. Finally, we investigated the ratio between mtDNA deletion level and mtDNA copy number in relation to BC risk. This measure represents the proportion of intact molecules relative to the total number of mtDNA (calculated as *ND1*/nuclear DNA content) molecules per subject.

Considering the number of statistical tests carried out in the present set of analyses, the threshold for statistical significance had to be corrected to avoid false-positive findings. Although it is not reasonable to assume that the three markers are independent, we preferred a conservative approach and thus considered the *p* value threshold of (0.05/3) < 0.017.

## Results

In the first step, we measured LTL and mtDNA copy number in 96 individuals belonging to the EPIC Heidelberg cohort, from whom a blood sample was collected at 2 time points approximately 15 years apart. Both measurements showed good stability over this 14- to 15-year period, with ICCs of 0.63 for LTL and 0.60 for mtDNA. Given the good reliability of the estimates, we then measured LTL and mtDNA copy number in 570 invasive BC cases and 538 control subjects recruited in the context of the prospective EPIC study. Four samples were included in all plates and analyzed in all assays for quality control. The highest observed coefficient of variation (CV) among these samples and across assays was 5.52%, and the average CV over all samples and all assays was 3.06%.

We also calculated CVs on the seven dilutions of the sample used as standard across genotyping plates and assays. The highest observed CV was 3.46%, and the average CV over all samples and all assays was 2.02%.

Regarding the distribution of LTL and mtDNA measures across different strata of risk covariates (Table 1), age was, as expected, inversely related to LTL (*p* = 1.66 × 10^− 11^) but was not associated with the mtDNA genome variables. Smoking did not show any association with the three markers measured, whereas BMI was significantly associated with mtDNA copy number, though with a modest effect size (correlation coefficient 0.009, *p* = 0.005). Similarly to a previous study [24], we also observed a modest but statistically significant, correlation between telomere length and mtDNA copy number (correlation coefficient 0.16, *p* = 7.3 × 10^− 4^), whereas there was no correlation between the other two markers (Additional file 1: Table S1).

**Table 1:** Association between selected markers and age, smoking, and body mass index

Marker	Age^a^		Smoking^b^		BMI^b^	
	Effect (SE)	*p* value	Effect (SE)	*p* value	Effect (SE)	*p* value
Telomere length	− 0.004 (0.001)	1.6 × 10^− 5^	− 0.002 (0.01)	0.84	− 0.0009 (0.002)	0.90
mtDNA copy number	0.002 (0.002)	0.33	0.04 (0.02)	0.04	0.009 (0.0032)	0.005
mtDNA deletions	0.0004 (0.0008)	0.65	− 0.01 (0.009)	0.10	0.001 (0.0014)	0.32

*BMI* Body mass index, *mtDNA* Mitochondrial DNA

^a^The analyses were adjusted for plate and case-control status

^b^The analyses were adjusted for age, plate, and case-control status

We observed that longer LTL was strongly associated with increased risk of BC when analyzing LTL as a categorical variable (OR 2.71, 95% CI 1.58–4.65, *p* = 3.07 × 10^− 4^ for highest vs. lowest quartile) and also as a continuous variable (OR 3.20, 95% CI 1.57–6.55, *p* = 1.41 × 10^− 3^). When we stratified the analysis by ER status of cancer, we observed that longer telomeres were associated with increased risk of both ER− BC (OR 2.12, 95% CI 1.04–4.31, *p* = 3.84 × 10^− 2^ for highest vs. lowest quartile) and ER+ BC (OR 2.55, 95% CI 1.10–5.86, *p* = 2.82 × 10^− 2^ for highest vs. lowest quartile). All results from the fully adjusted analyses on LTL and BC risk are shown in Table 2. (Minimally adjusted models are shown in Additional file 2: Table S2.)

**Table 2:** Associations between leukocyte telomere length and breast cancer risk

Stratum	Relative LTL^a^	Controls	Cases	OR	95% CI	*p* value
Overall	Quartile 1 (0.36–0.63)	134	81	Reference	–	–
	Quartile 2 (0.63–0.75)	126	102	1.60	(1.04–2.46)	3.17E-02
	Quartile 3 (0.75–0.93)	134	172	2.28	(1.46–3.57)	2.89E-04
	Quartile 4 (0.93–1.94)	139	215	2.71	(1.58–4.65)	3.07E-04
	Continuous variable	533	570	3.20	(1.57–6.55)	1.41E-03
ER+	Quartile 1 (0.36–0.63)	134	41	Reference	–	–
	Quartile 2 (0.63–0.75)	126	45	1.59	(0.88–2.89)	1.25E-01
	Quartile 3 (0.75–0.93)	134	77	2.97	(1.54–5.72)	1.11E-03
	Quartile 4 (0.93–1.94)	139	86	2.55	(1.10–5.86)	2.82E-02
	continuous variable	533	249	3.33	(1.17–9.46)	2.38E-02
ER−	Quartile 1 (0.36–0.63)	134	28	Reference	–	–
	Quartile 2 (0.63–0.75)	126	40	1.25	(0.65–2.44)	5.05E-01
	Quartile 3 (0.75–0.93)	134	79	1.67	(0.88–3.16)	1.19E-01
	Quartile 4 (0.93–1.94)	139	104	2.12	(1.04–4.31)	3.84E-02
	Continuous variable	533	251	2.33	(0.88–6.15)	8.92E-02

*ER* Estrogen receptor, *LTL* Leukocyte telomere length

^a^Analyses are adjusted for age, body mass index, smoking, center of origin, and plate

For mtDNA copy numbers, we did not observe any differential association between cases and controls when considering quartiles. Also, the stratified analysis (ER+/ER−) did not show any statistically significant differences between cases and controls (Table 3). The results of the minimally adjusted models are shown in Additional file 3: Table S3. However, we included in the analysis the ratio between mtDNA deletion level and mtDNA copy (*ND4*/*ND1* divided by *ND1*/nuclear genome). When comparing this measure across cases and controls, we observed an increase in risk with increased number of copies. This difference was statistically significant only for ER+ BC. When analyzed as a categorical variable, unconditional logistic regression models showed a significant association of ER+ BC with mtDNA integrity (OR 2.47, 95% CI 1.05–5.80, for highest vs. lowest quartile) (Table 4). The results of the minimally adjusted models are shown in Additional file 4: Table S4. Finally when analyzing the score (computing the aggregate effect of mtDNA copy number and LTL) as a continuous variable, we observed an increased risk for individuals with a high score compared with individuals with a low score (OR 1.20, 95% CI 1.05–1.37, *p* = 0.009). This trend was present in all the strata but was significant only in the analysis considering ER+ and ER− together (Table 5).

**Table 3:** Associations between mitochondrial DNA copy number and breast cancer risk

Stratum	mtDNA copy number^a^	Controls	Cases	OR	95% CI	*p* value
Overall	Quartile 1 (0.11–0.21)	132	117	Reference	–	–
	Quartile 2 (0.21–0.29)	132	148	1.48	(1.00–2.20)	5.24E-02
	Quartile 3 (0.29–0.47)	125	122	1.30	(0.81–2.11)	2.77E-01
	Quartile 4 (0.47–1.52)	133	161	0.93	(0.53–1.66)	8.18E-01
	Continuous variable	522	548	0.97	(0.66–1.42)	8.75E-01
ER+	Quartile 1 (0.11–0.21)	132	68	Reference	–	–
	Quartile 2 (0.21–0.29)	132	94	1.43	(0.85–2.39)	1.77E-01
	Quartile 3 (0.29–0.47)	125	65	1.23	(0.65–2.32)	5.22E-01
	Quartile 4 (0.47–1.52)	133	18	0.46	(0.18–1.13)	8.95E-02
	Continuous variable	522	245	0.67	(0.36–1.25)	2.11E-01
ER−	Quartile 1 (0.11–0.21)	132	41	Reference	–	–
	Quartile 2 (0.21–0.29)	132	42	1.33	(0.74–2.38)	3.43E-01
	Quartile 3 (0.29–0.47)	125	35	0.90	(0.43–1.91)	7.91E-01
	Quartile 4 (0.47–1.52)	133	140	0.94	(0.44–2.02)	8.81E-01
	Continuous variable	522	258	1.04	(0.64–1.69)	8.79E-01

*ER* Estrogen receptor, *mtDNA* Mitochondrial DNA

^a^Analyses are adjusted for age, body mass index, smoking, center of origin, and plate

**Table 4:** Associations between mitochondrial DNA integrity (ND4/ND1 divided by ND1/nuclear genome) and breast cancer risk

Stratum	mtDNA integrity^a^	Controls	Cases	OR	95% CI	*p* value
Overall	Quartile 1 (0.34–0.90)	129	152	Reference		
	Quartile 2 (0.90–1.00)	126	102	1.06	(0.68–1.66)	0.79
	Quartile 3 (1.00–1.11)	133	142	1.56	(0.95–2.56)	0.08
	Quartile 4 (1.11–2.59)	130	142	1.20	(0.69–2.08)	0.52
	Continuous variable	519	539	1.07	(0.75–1.52)	0.73
ER+	Quartile 1 (0.34–0.90)	129	20	Reference	–	–
	Quartile 2 (0.90–1.00)	126	50	1.61	(0.78–3.35)	0.20
	Quartile 3 (1.00–1.11)	133	79	2.59	(1.19–5.66)	0.02
	Quartile 4 (1.11–2.59)	130	89	2.47	(1.05–5.80)	0.04
	Continuous variable	519	238	1.54	(0.87–2.72)	0.14
ER−	Quartile 1 (0.34–0.90)	129	128	Reference	–	–
	Quartile 2 (0.90–1.00)	126	40	0.98	(0.55–1.74)	0.94
	Quartile 3 (1.00–1.11)	133	47	1.22	(0.62–2.39)	0.56
	Quartile 4 (1.11–2.59)	130	41	0.96	(0.46–2.00)	0.92
	Continuous variable	519	256	0.99	(0.63–1.54)	0.95

*ER* Estrogen receptor, *mtDNA* Mitochondrial DNA

^a^Analyses are adjusted for age, body mass index, smoking, center of origin, and plate

**Table 5:** Associations between a biomarker score created from leukocyte telomere length and mitochondrial DNA copy number and breast cancer risk

Stratum	Controls	Cases	OR	95% CI	*p* value
Overall	522	548	1.20	(1.05–1.37)	0.009
ER+	522	245	1.18	(0.96–1.45)	0.11
ER−	522	258	1.16	(0.98–1.38)	0.09

*ER* Estrogen receptor

## Discussion

Given their central role in several essential functions of the eukaryotic cell, mitochondria—and in particular mitochondrial mutations—have been extensively investigated in relation to human cancer. A growing number of somatic mitochondrial mutations have been implicated in several cancer types, including BC [50, 51]. In addition, the common germline variability in the mitochondrial genome (deletions, mitochondrial single-nucleotide polymorphisms, and resulting mitochondrial haplogroups) has been associated with risk of various cancers, such as of the lung, pancreas, and breast [50, 52–54]. Several studies have suggested that, to counterbalance mitochondrial dysfunction caused by genetic mutations, the eukaryotic cell increases their number. Mitochondrial copy number has been proposed as a risk marker for various cancer types [5–7, 9, 10, 12, 13]. In a small exploratory study on BC, Shen and colleagues reported an association between high mtDNA copy number and increased risk of the disease [5]. This association was subsequently replicated in two independent studies focused primarily on ER+ BC [9, 13] and again by Shen and colleagues [18].

Telomeres are specialized structures that adorn the telomeric end of the eukaryotic chromosomes and are essential for the correct segregation of chromosomes to daughter cells [55] as well as for the control of chromosomal stability and regulation of cell growth [56–58]. In the past several years, there has been an overwhelming number of studies aimed at understanding the role of telomere length, measured in blood, in relation to cancer risk and progression. The results, however, are often inconclusive, with some articles reporting an association between increased risk of developing various cancer types and longer telomeres and others reporting the opposite, no association at all, or even a nonlinear dose-response association with increased risks only at the two extremes of the LTL distribution (very short and very long LTL) [26–29].

LTL and mtDNA copy number have largely been examined as independent contributors to cancer risk; yet, there is emerging evidence that the markers are linked biologically or at least that measuring them together increases the efficiency of the estimation of their effect. For example, Bao and colleagues [59] analyzed LTL and mtDNA as progression markers for hepatocellular carcinoma and observed that the combination of the two was a better indicator than each of the two separately. Moreover, it has been demonstrated that LTL and mtDNA copy number are correlated in intestinal gastric cancer [32]. Recently, an association between mitochondrial dysfunction and telomere length attrition has been suggested, and a correlation between LTL and mtDNA has been observed by several authors [24, 31, 60]. Both telomere length and mitochondrial function have been proposed as markers of aging (although the evidence is much stronger for telomeres). Mice null for either the telomerase reverse transcriptase (Tert) or telomerase RNA component (Terc) gene had marked repression of peroxisome proliferator-activated receptor-γ, coactivator 1 alpha and beta, and the downstream network. Consistent with peroxisome proliferator-activated receptors as master regulators of mitochondrial physiology and metabolism, telomere dysfunction, including shortening, was associated with impaired mitochondrial biogenesis and function, decreased gluconeogenesis, cardiomyopathy, and increased ROS [25].

Given the mounting evidence of possible combined associations of cancer risk with LTL and mtDNA, we sought to investigate, for the first time in the same study, the two markers with the addition of mtDNA deletion in order to gain a more comprehensive picture of their involvement in BC risk. Given the large number of caveats linked to these measurements as a first preliminary step, we wanted to determine how stable the measurements were. To do so, we isolated DNA from blood samples taken 15 years apart from 96 individuals. We observed a very good stability over time for both LTL (ICC 0.63) and mtDNA copy number (ICC 0.60). These values probably underestimate the real correlations because the starting material was not exactly the same: The DNA collected at baseline was isolated from buffy coat, whereas for the samples collected after 15 years, the extraction was done starting from whole blood.

In the cross-sectional analysis, our results suggest a clear association between longer telomeres and increased risk of BC independent of ER status. The association between longer telomeres and increased BC risk is biologically plausible because longer LTL may be a marker of an actively reproducing cell and therefore a cell that keeps dividing and that is exposed to an increased risk of acquiring tumor-causing mutations. The association between longer or shorter LTL and cancer risk could also be tumor-specific and may reflect the complex interaction between the genetic determination of telomere length and the environmental risk factors specific for each cancer type.

The analyses of our data did not show any strong association between mtDNA, when analyzed alone, and BC risk, contrary to what has been observed by others [5, 9, 13, 18]. Authors of two recent meta-analyses investigated the role of mtDNA copy number in a large number of studies across different tumors and highlighted the great heterogeneity of the mtDNA effect in different cancer types and a null overall effect [61, 62]. Interestingly, also the four studies in which researchers reported the association with BC showed a high degree of heterogeneity, possibly explaining the difference between those studies and our findings. However, when normalizing for mitochondrial deletions and using the mitochondrial integrity variable, we did observe an increase in risk with a higher number of functional copies. This association is biologically plausible because an increase in mtDNA copy number may reflect increased oxidative stress that in turn may increase damage levels, fuel inflammation [63], and finally increase the risk of developing BC. One of the novelties of our report is the introduction of a measure of mitochondrial genome integrity and the possibility of observing the effect of functional mitochondrial copy number in cancer risk instead of just measuring the effect mtDNA copy number regardless of their status. Given that when considering mtDNA copy number alone we did not see any association with BC risk, we hypothesize that this “correction,” at least in part, could explain the differences and heterogeneity observed across previous reports. Analyzing the score of mtDNA and LTL, we observed an association with increased risk for the individuals with a high score; however, this probably reflects the association between LTL and BC risk.

This study has several clear strengths, one being its prospective design, which, to a certain extent, may reduce the possibility of reverse causation, which is a critical point when looking for the relationship between LTL, mtDNA, and cancer risk. A second strength is the possibility of avoiding possible confounders by adjusting for all known potential confounding factors on which information was available, such as smoking, BMI, and alcohol consumption. In addition, our data suggest that our calculations of the LTL and mtDNA ratios are unlikely to be heavily affected by sample handling or technical errors, given that even at 15 years apart the measurements remained comparable. A possible limitation of the present study is the relatively small sample size, which is partially counterbalanced, however, by the rather large number of ER− BC cases.

## Conclusions

We observed a very good correlation between the markers across a period of 15 years, highlighting how sample handling is crucial for this kind of analysis. Moreover, we also observed a strong and consistent association between longer telomere length and increased BC risk, and additionally, we found a novel association between the mitochondrial genome stability and ER+ BC risk.

## Additional files

Additional file 1: **Table S1.** Correlation between the biomarkers in control subjects. (DOCX 13 kb)

Additional file 2: **Table S2.** Associations between LTL and BC risk. (DOCX 15 kb)

Additional file 3: **Table S3.** Associations between mtDNA copy number and BC risk. (DOCX 15 kb)

Additional file 4: **Table S4.** Associations between mtDNA integrity (*ND4*/*ND1* divided by *ND1*/nuclear genome) and BC risk. (DOCX 15 kb)

## Abbreviations

BC: Breast cancer
BMI: Body mass index
CV: Coefficient of variation
EPIC: European Prospective Investigation into Cancer and Nutrition
ER: Estrogen receptor
ICC: Intraclass correlation
ICD-10: International Classification of Diseases, Tenth Revision
LTL: Leukocyte telomere length
mtDNA: Mitochondrial DNA
ND: Nicotinamide adenine dinucleotide dehydrogenase
