# Supplementary material for: Mitochondrial DNA copy number variation, leukocyte telomere length, and breast cancer risk in the European Prospective Investigation into Cancer and Nutrition (EPIC) study
Source: Breast Cancer Res. 2018 Apr 17;20:29. doi: 10.1186/s13058-018-0955-5 (PMC5905156; doi:10.1186/s13058-018-0955-5)
Supplement: Supplementary file 1 — Table S1. Correlation between the biomarkers in control subjects. (DOCX 13 kb) [file 13058_2018_955_MOESM1_ESM.docx]

**Supplementary table 1**. Correlation between the biomarkers in control subjects.

| Biomarkers | Correlation coeff. | P-value |
| --- | --- | --- |
| Telomere length, MtDNA copy number | 0.16 | 7.3x10^-4^ |
| Telomere length, MtDNA deletion | 0.05 | 0.2 |
| MtDNA copy number, MtDNA deletion | -0.08 | 0.055 |

The table shows Pearson’s correlation coefficients and p-values.
