# Supplementary material for: Mitochondrial DNA copy number variation, leukocyte telomere length, and breast cancer risk in the European Prospective Investigation into Cancer and Nutrition (EPIC) study
Source: Breast Cancer Res. 2018 Apr 17;20:29. doi: 10.1186/s13058-018-0955-5 (PMC5905156; doi:10.1186/s13058-018-0955-5)
Supplement: Supplementary file 2 — Table S2. Associations between LTL and BC risk. (DOCX 15 kb) [file 13058_2018_955_MOESM2_ESM.docx]

**Supplementary table 2**. Associations between LTL and BC risk.

|  |  |  |  | **Minimally adjusted**  **OR (95% CI)^a^** |  |  |
| --- | --- | --- | --- | --- | --- | --- |
| **Stratum** | **Relative LTL** | **Controls** | **Cases** |  | **P_value_** |  |
| Overall | Quartile 1 (0.36-0.63) | 134 | 81 | - | - |  |
|  | Quartile 2 (0.63-0.75) | 126 | 102 | 1.41 (0.95,2.10) | 9.11E-02 | |
|  | Quartile 3 (0.75-0.93) | 134 | 172 | 1.95 (1.28,2.97) | 1.79E-03 | |
|  | Quartile 4 (0.93-1.94) | 139 | 215 | 2.31 (1.38,3.88) | 1.52E-03 | |
|  | continuous variable | 533 | 570 | 2.28 (1.17,4.43) | 1.52E-02 | |
| ER+ | Quartile 1 (0.36-0.63) | 134 | 41 | - | - | |
|  | Quartile 2 (0.63-0.75) | 126 | 45 | 1.23 (0.73,2.06) | 4.38E-01 | |
|  | Quartile 3 (0.75-0.93) | 134 | 77 | 2.04 (1.16,3.58) | 1.29E-02 | |
|  | Quartile 4 (0.93-1.94) | 139 | 86 | 1.73 (0.82,3.64) | 1.50E-01 | |
|  | continuous variable | 533 | 249 | 1.62 (0.67,3.9) | 2.85E-01 | |
| ER- | Quartile 1 (0.36-0.63) | 134 | 28 | - | - | |
|  | Quartile 2 (0.63-0.75) | 126 | 40 | 1.37 (0.72,2.62) | 3.36E-01 | |
|  | Quartile 3 (0.75-0.93) | 134 | 79 | 1.75 (0.93,3.27) | 8.13E-02 | |
|  | Quartile 4 (0.93-1.94) | 139 | 104 | 2.28 (1.14,4.57) | 2.03E-02 | |
|  | continuous variable | 533 | 251 | 2.15 (0.87,5.35) | 9.79E-01 | |

^a^ the minimally adjusted models account for study center, age and plate
