# Supplementary material for: Mitochondrial DNA copy number variation, leukocyte telomere length, and breast cancer risk in the European Prospective Investigation into Cancer and Nutrition (EPIC) study
Source: Breast Cancer Res. 2018 Apr 17;20:29. doi: 10.1186/s13058-018-0955-5 (PMC5905156; doi:10.1186/s13058-018-0955-5)
Supplement: Supplementary file 3 — Table S3. Associations between mtDNA copy number and BC risk. (DOCX 15 kb) [file 13058_2018_955_MOESM3_ESM.docx]

**Supplementary table 3**. Associations between mtDNA copy number and BC risk.

|  |  |  |  | **Minimally adjusted**  **OR (95% CI)^a^** |  |
| --- | --- | --- | --- | --- | --- |
| **Stratum** | **mtDNA copy number** | **Controls** | **Cases** |  | **P_value_** |
| Overall | Quartile 1 (0.11-0.21) | 132 | 117 | - | - |
|  | Quartile 2 (0.21-0.29) | 132 | 148 | 1.47 (0.99,2.17) | 5.38E-02 |
|  | Quartile 3 (0.29-0.47) | 125 | 122 | 1.20 (0.75,1.91) | 4.56E-01 |
|  | Quartile 4 (0.47-1.52) | 133 | 161 | 0.93 (0.53,1.63) | 7.90E-01 |
|  | continuous variable | 522 | 548 | 0.97 (0.67,1.42) | 8.85E-01 |
| ER+ | Quartile 1 (0.11-0.21) | 132 | 68 | - | - |
|  | Quartile 2 (0.21-0.29) | 132 | 94 | 1.40 (0.86,2.28) | 1.71E-01 |
|  | Quartile 3 (0.29-0.47) | 125 | 65 | 1.22 (0.69,2.18) | 4.96E-01 |
|  | Quartile 4 (0.47-1.52) | 133 | 18 | 0.59 (0.26,1.34) | 2.08E-01 |
|  | continuous variable | 522 | 245 | 0.79 (0.46,1.38) | 4.21E-01 |
| ER- | Quartile 1 (0.11-0.21) | 132 | 41 | - | - |
|  | Quartile 2 (0.21-0.29) | 132 | 42 | 1.24 (0.71,2.16) | 4.47E-01 |
|  | Quartile 3 (0.29-0.47) | 125 | 35 | 0.82 (0.41,1.64) | 5.79E-01 |
|  | Quartile 4 (0.47-1.52) | 133 | 140 | 0.92 (0.46,1.84) | 8.12E-01 |
|  | continuous variable | 522 | 258 | 1.04 (0.66,1.63) | 8.64E-01 |

^a^ the minimally adjusted models account for study center, age and plate
