# Supplementary material for: Mitochondrial DNA copy number variation, leukocyte telomere length, and breast cancer risk in the European Prospective Investigation into Cancer and Nutrition (EPIC) study
Source: Breast Cancer Res. 2018 Apr 17;20:29. doi: 10.1186/s13058-018-0955-5 (PMC5905156; doi:10.1186/s13058-018-0955-5)
Supplement: Supplementary file 4 — Table S4. Associations between mtDNA integrity (ND4/ND1 divided by ND1/nuclear genome) and BC risk. (DOCX 15 kb) [file 13058_2018_955_MOESM4_ESM.docx]

**Supplementary table 4**. Associations between mtDNA integrity (ND4/ND1 divided by ND1/nuclear genome), and BC risk.

|  |  |  |  | **Minimally adjusted**  **OR (95% CI)^a^** |  |
| --- | --- | --- | --- | --- | --- |
| **Stratum** | **mtDNA DNA integrity** | **Controls** | **Cases** |  | **P_value_** |
| Overall | Quartile 1 (0.34-0.90) | 129 | 152 | - | - |
|  | Quartile 2 (0.90-1.00) | 126 | 102 | 1.29 (0.83,2.03) | 2.60E-01 |
|  | Quartile 3 (1.00-1.11) | 133 | 142 | 1.71 (1.05,2.77) | 3.02E-02 |
|  | Quartile 4 (1.11-2.59) | 130 | 142 | 1.59 (0.93,2.70) | 9.02E-02 |
|  | continuous variable | 519 | 539 | 1.10 (0.43,2.80) | 8.43E-01 |
| ER+ | Quartile 1 (0.34-0.90) | 129 | 20 | - | - |
|  | Quartile 2 (0.90-1.00) | 126 | 50 | 1.47 (0.79,2.75) | 2.27E-01 |
|  | Quartile 3 (1.00-1.11) | 133 | 79 | 2.44 (1.23,4.84) | 1.05E-02 |
|  | Quartile 4 (1.11-2.59) | 130 | 89 | 2.30 (1.07,4.95) | 3.23E-02 |
|  | continuous variable | 519 | 238 | 2.09 (0.50,8.82) | 3.16E-01 |
| ER- | Quartile 1 (0.34-0.90) | 129 | 128 | - | - |
|  | Quartile 2 (0.90-1.00) | 126 | 40 | 1.28 (0.70,2.34) | 4.25E-01 |
|  | Quartile 3 (1.00-1.11) | 133 | 47 | 1.19 (0.63,2.24) | 6.01E-01 |
|  | Quartile 4 (1.11-2.59) | 130 | 41 | 1.12 (0.56,2.23) | 7.42E-01 |
|  | continuous variable | 519 | 256 | 0.64 (0.21,2.01) | 4.49E-01 |

^a^ the minimally adjusted models account for study center, age and plate
